# Facile Synthesis of Free-Standing NiO/MnO_2_ Core-Shell Nanoflakes on Carbon Cloth for Flexible Supercapacitors

**DOI:** 10.1186/s11671-017-1939-6

**Published:** 2017-03-07

**Authors:** Shuang Xi, Yinlong Zhu, Yutu Yang, Shulan Jiang, Zirong Tang

**Affiliations:** 1 School of Mechanical and Electronic Engineering, Nanjing Forestry University, Nanjing, 210037 China; 2 Tribology Research Institute, Southwest Jiaotong University, Chengdu, 610031 China; 3 Wuhan National Laboratory for Optoelectronics, Huazhong University of Science and Technology, Wuhan, 430074 China

**Keywords:** NiO, MnO_2_, Nanoflakes, Flexible, Supercapacitors

## Abstract

Free-standing NiO/MnO_2_ core-shell nanoflake structure was deposited on flexible carbon cloth (CC) used as electrode for high-performance supercapacitor (SC). The NiO core was grown directly on CC by hydrothermal process and the following annealing treatment. MnO_2_ thin film was then covered on NiO structures via a self-limiting process in aqueous solution of 0.5 M KMnO_4_ and 0.5 M Na_2_SO_4_ with a carbon layer serving as the sacrificial layer. Both the core and shell materials are good pseudocapacitive materials, the compounds of binary metal oxides can provide the synergistic effect of all individual constituents, and thus enhance the performance of SC electrode. The obtained CC/NiO/MnO_2_ heterostructure was directly used as SC electrodes, showing an enhanced electrochemical performance including areal capacitance of 316.37 mF/cm^2^ and special gravimetric capacitance of 204.3 F/g at the scan rate of 50 mV/s. The electrode also shows excellent cycling stability, which retains 89% of its initial discharge capacitance after 2200 cycles with >97% Coulombic efficiency. The synthesized binder-free hierarchical composite electrode with superior electrochemical properties demonstrates enormous potential in the application of flexible SCs.

## Background

Flexible and light-weight energy storage devices have attracted increasing attention due to the proliferating demand for wearable and portable consumer electronics in the modern society, while the conventional capacitors and batteries are too bulky in size and heavy in weight [1–5]. Among various flexible energy storage devices, supercapacitors (SCs), also named electrochemical capacitors (ECs), have gain ample attention, as they can provide an instantaneous higher power density, fast charging, long life cycles when compared to flexible batteries [6]. Flexible supercapacitors based on storage mechanisms are classified into two major classes: electrical double-layer capacitors (EDLCs) using carbon materials and Faradic redox reaction pseudocapacitors using conducting polymers and transition metal oxides [7]. Compared with EDLCs, pseudocapacitors possess higher specific capacitance attributed to their fast and reversible redox reaction, so most of recent researches of SCs have focused on the development of transition metal oxides with variable valence which attributes to the pseudocapacitance generation [8].

As we all know, ruthenium oxide (RuO_2_) is the best pseudocapacitance material mainly because of its high specific capacitance [9, 10]. However, the expensive cost for mass production hinders its widely application, and thus, some alternatives such as MnO_2_ [11–13], NiO [14], Co_3_O_4_ [15], CuO [16], and V_2_O_5_ [17] with similar pseudocapacitive behavior emerge. Among these candidates, manganese oxides have variety of advantages apart from their excellent electrochemical properties, such as low cost, environmentally friendly nature, and abundance, making it one of the most attractive electrode materials for pseudocapacitors [18, 19]. However, its inherent poor electrical conductivity as well as the short diffusion depth around 20 nm, makes electrons can just transport near the surface of electroactive material, while the underneath parts are blocked for participating in the electrochemical process [20]. As for a SC electrode, the specific capacitance only arises from the surface of the bulk material and, thus, a less satisfied SC can be obtained. What’s more, in traditional fabrication process for SCs, the active materials are often combined to the current collector through the use of polymer binder, which can produce “dead volume” in active materials and thus result in lower capacitance [21]. To address these issues, a feasible strategy is to directly grow well-designed integrated architectures combining of two or more materials with high redox electroactivity into one ordered nanostructure onto conducting substrates as binder-free electrodes [22]. In this model, electrode binder is avoided to enhance the electron collection efficiency and the hierarchical active nanostructures might produce synergistic effect to improve electrochemical properties.

Liu et al. [23] fabricated rod-like NiO core with sphere-like MnO_2_ shell on the Ni foam, getting a high areal capacitance of 3.584 F/cm^2^ at a current density of 5 mA/cm^2^. Fan [24] reported the direct synthesis of MnO_2_-NiO nanoflake-assembled tubular array on stainless steel substrate to function as pseudocapacitor electrode, which has shown good rate performance and cycle life. Zhonglin Wang [8] reported hydrogenated ZnO/MnO_2_ core-shell nanocables integrated on carbon cloth for flexible all-solid-state supercapacitors and demonstrated its application in stand-alone self-powered systems.

Inspired by these advances, we demonstrated a facile route for the construction of NiO/MnO_2_ core-shell heterostructures on three dimensional carbon cloth (CC) as a flexible electrode. NiO has the advantages of natural abundance, low cost, environmental friendliness, and high theoretical capacity (718 mAh/g). Moreover, it’s easy for NiO to form free-standing nanostructures which can be used as a backbone to support active electrode materials MnO_2_ forming 3D hierarchical hybrid nanostructures for high-performance SCs. Both the core and shell materials are good pseudocapacitive materials, the compounds of binary metal oxides can provide the synergistic effect of all individual constituents and thus enhance the performance of SC electrode. The core-shell nanoflakes are strongly supported on CC, avoiding the use of polymer binder, to reduce the “dead volume” in active material. To the best of our knowledge, the NiO/MnO_2_ compositing nanostructure-based flexible electrodes have not yet been reported. Impressively, the obtained NiO/MnO_2_ core-shell heterostructure integrated on CC exhibits large areal capacitance of 286 mF/cm^2^ at current density of 0.5 mA/cm^2^, desirable rate performance and cycling stability in 1 M Na_2_SO_4_ solution.

## Materials and Methods

### Materials

CC with hydrophilic surfaces was purchased from Taiwan CeTech Co. Ltd. All chemicals were of analytical grade. 1-Ethyl-3-methylimidazolium dicyanamide (EMID) was purchased from Adamas Reagent Co., Ltd. NiCl_2_•6H_2_O, CO(NH_2_)_2_ , NH_4_F, KMnO_4_, and Na_2_SO_4_ were purchased from Sinopharm Chemical Reagent Co. Ltd., (China). All chemicals were used as received without any further purification. Deionized (DI) water (18.2 MU cm) from Milli-Q was used throughout the entire experiment, and all aqueous solutions were prepared with ultrapure water.

### Synthesis of NiO Nanosheets on CC

NiO nanoflakes were integrated onto CC by hydrothermal method based on our previous report [25]. Briefly, a precursor solution was prepared with 5 mM NiCl_2_•6H_2_O, 25 mM CO(NH_2_)_2_, and 10 mM NH_4_F dissolved in 100 ml deionized water. After stirring for 30 min, the as-obtained solution was transferred into Teflon-lined stainless autoclave, and then a piece of well-cleaned CC was dipped in the precursor solution. The autoclave was sealed and maintained at 120 °C for 4 h in an electric oven. After cooling down to the room temperature naturally, the sample was taken out, rinsed with ethanol and distilled water for several times, and dried in a vacuum oven at 60 °C for 12 h. This process led to the coating of nickel hydroxide onto the substrate. An additional annealing process was carried out with a quartz tube at 350 °C for 2 h in air atmosphere to transform nickel hydroxide into NiO as well as to improve the mechanical and electrical adhesion between nanosheets and CC.

### Synthesis of NiO/MnO_2_ Heterostructure Composite on CC

To obtain NiO/MnO_2_ core-shell heterostructures, a carbon layer was first wrapped on the surface of NiO nanosheets to serve as a sacrificial reductant by dipping EMID onto the as-prepared sample followed with thermal annealing (air atmosphere, 450 °C, 100 min). After that, the obtained NiO/carbon composite was immersed into an aqueous solution with equal volume of 0.5 M KMnO_4_ and 0.5 M Na_2_SO_4_ for 8 h at room temperature, and the core-shell NiO/MnO_2_ heterostructure was obtained on CC via a self-limiting process [8, 26]. Then, the sample was cleaned several times using deionized water.

### Characterization

The structural properties of the samples were characterized by field emission scanning electron microscopy (FESEM, Hitachi, S-4800, Japan), high-resolution transmission electron microscope (HRTEM, FEI-F20) equipped with energy-dispersive x-ray spectrometry (EDS) and selected area electron diffraction (SAED), X-ray diffraction (XRD, Bruker D8 Advance) with Cu-Ka radiation (1.5418°A) operating at 40 kV, 100 mA. X-ray photoelectron spectroscopy (XPS) was carried out at room temperature in ESCALAB 250 system.

### Electrochemical Measurement

The NiO/MnO_2_ compositing nanoflake modified CC electrode was evaluated for a high-performance SCs by the three-electrode system in 1 M KOH aqueous solution. The three-electrode system was constructed using the obtained sample as the working electrode, a saturated calomel electrode (SCE) as the reference electrode, and a Pt foil as the counter electrode. Cyclic voltammetry (CV) and electrochemical impedance spectroscopy (EIS) tests were conducted on an Autolab work station (PGSTAT-302 N, Eco Echemie B.V. Company, Utrecht, Netherlands). For comparison, CC/NiO electrode was also fabricated, and its electrochemical performance was tested at the same conditions as those for CC/NiO/MnO_2_ composite by CV technique. Galvanostatic charging/discharging and cycling tests were conducted using a battery measurement system (LAND CT2001A, Wuhan LAND Electronics, Wuhan, China).

## Results and Discussion

**Fig. 1:**
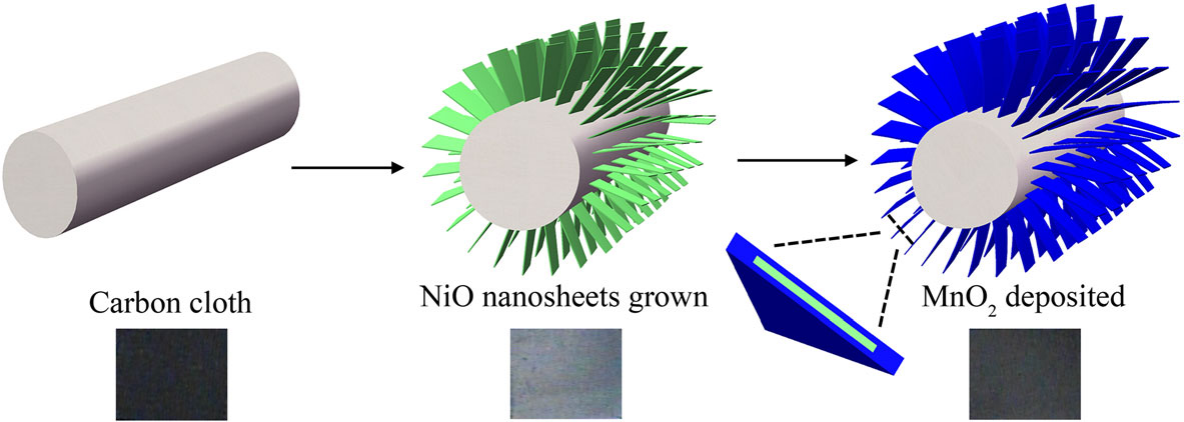
Typical synthesizing process of the integrating structures, with the photographic images of CC after nanostructure integrated showing underneath

The general electrode fabrication procedure is schematically illustrated in Fig. 1. Initially, free-standing NiO nanosheets were grown on CC using a hydrothermal synthesis and subsequent annealing treatment. The inset figure shows the optical images of the CC before and after NiO growth. It can be observed that the pure CC is with deep black color while the CC/NiO sample is with light green color. After MnO_2_ layer was deposited on the assembly, the color of the sample became dark gray.

**Fig. 2:**
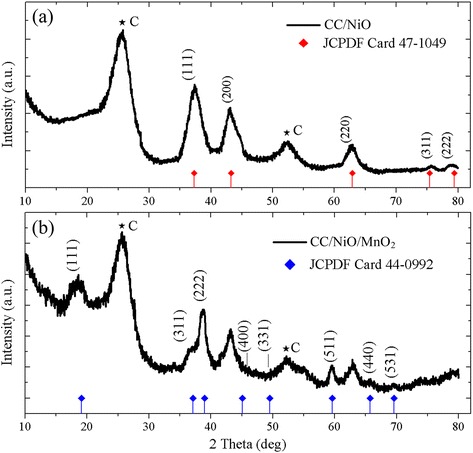
XRD pattern of as-prepared composite. **a** CC/NiO and **b** CC/NiO/MnO_2_

The composition and phase purity of the as-prepared samples were examined by X-ray diffraction (XRD), showing in Fig. 2. As shown in Fig. 2a, the diffraction pattern exhibits the characteristic peaks of face-centered cubic NiO at 2θ = 37.3° (111), 43.3° (200), 62.9° (220), 75.4° (311), 79.4° (222), which are in accordance with the standard spectrum (JCPDS, Card No. 47-1049). Besides, the observed peaks at 26 and 54° can be ascribed to the carbon cloth substrate, which can also be observed in the XRD pattern of pure CC [25]. The XRD results affirm that NiO has been obtained through the aforementioned procedures. Then, the liquid EMID was added to the prepared sample dropwise and subsequently annealed in air atmosphere. An amorphous carbon layer was formed, and it can serve as a reducing agent for the deposition of MnO_2_ via the following redox reaction at room temperature

$$ 4 M n{O}_4^{-}+3 C+{H}_2 O=4 M n{O}_2+ C{O}_3^{2-}+2 H C{O}_3^{-} $$

In our case, the carbon layer uniformly coated on NiO nanostructures acts as a sacrificial template to direct the 3D interfacial reaction. Figure 2b shows the six characteristic peaks at 19, 37, 39, 59, 65 and 69° indicating the presence of MnO_2_. It can be seen that these MnO_2_ peaks are broad and unclear, which indicate the amorphous nature of the products. The XRD patterns for MnO_2_ corresponding to crystalline α-MnO_2_ are in accordance with the standard spectrum (JCPDS, Card No. 44-0992), where the other two peaks at 45° (400) and 49° (331) are too weak to be seen. Furthermore, the XRD spectrum in Fig. 2b also exhibits the diffraction peaks of NiO except for those of MnO_2_, revealing that the MnO_2_/NiO compositing structure has been obtained.

**Fig. 3:**
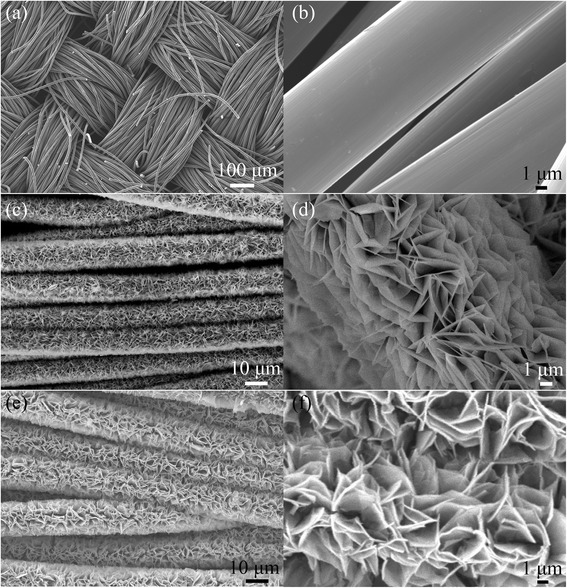
SEM of **a, b** primary CC. **c, d** CC/NiO nanosheets. **e**, **f** CC integrated with NiO/MnO_2_ nanostructures, with the whole images showing at the *left* and enlarged SEM images showing at the *right side*

The SEM image of the obtained structure is shown in Fig. 3, and the primary CC is demonstrated as Fig. 3a, b. It illustrates that the CC possesses ordered texture structure, where each carbon fiber presents smooth surface with a uniform diameter of around 10 μm. The morphology of the as-prepared CC/NiO nanosheets shown in Fig. 3c reveals that the ordered woven structure of the CC still remains. A close SEM examination demonstrates the uniform coverage of NiO nanosheets on CC surfaces (Fig. 3d), and the well-established texture structure of the NiO nanosheets grown perpendicularly on the carbon fibers. The nanosheets are interconnected with each other, which can not only create porous nanostructure with abundant open space and electroactive surface but also buffer the volume change resulted from both external bending stress and repeated ion insertion-extraction. The free space between nanosheets will be beneficial for the diffusion of electrolyte into the electrode, leading to reduced diffusion lengths of ions.

As shown in Fig. 3e, f, the NiO nanosheets became a little bit thicker and larger in size after depositions of the carbon layer and a subsequent deposition of MnO_2_ layer; moreover, the nanoflakes’ morphology was slightly distorted, which might be caused by the long-time redox reaction in solution. However, the obtained NiO/MnO_2_ nanoflakes are still well aligned on the substrate on a large scale, as displayed in Fig. 3e. It should be pointed out that the second 3D interfacial reaction occurs at the interface between the pre-grown NiO nanosheets, and the post-grown MnO_2_ nanoflakes have the same “roots”, which would make the hybrid structure highly integrated.

**Fig. 4:**
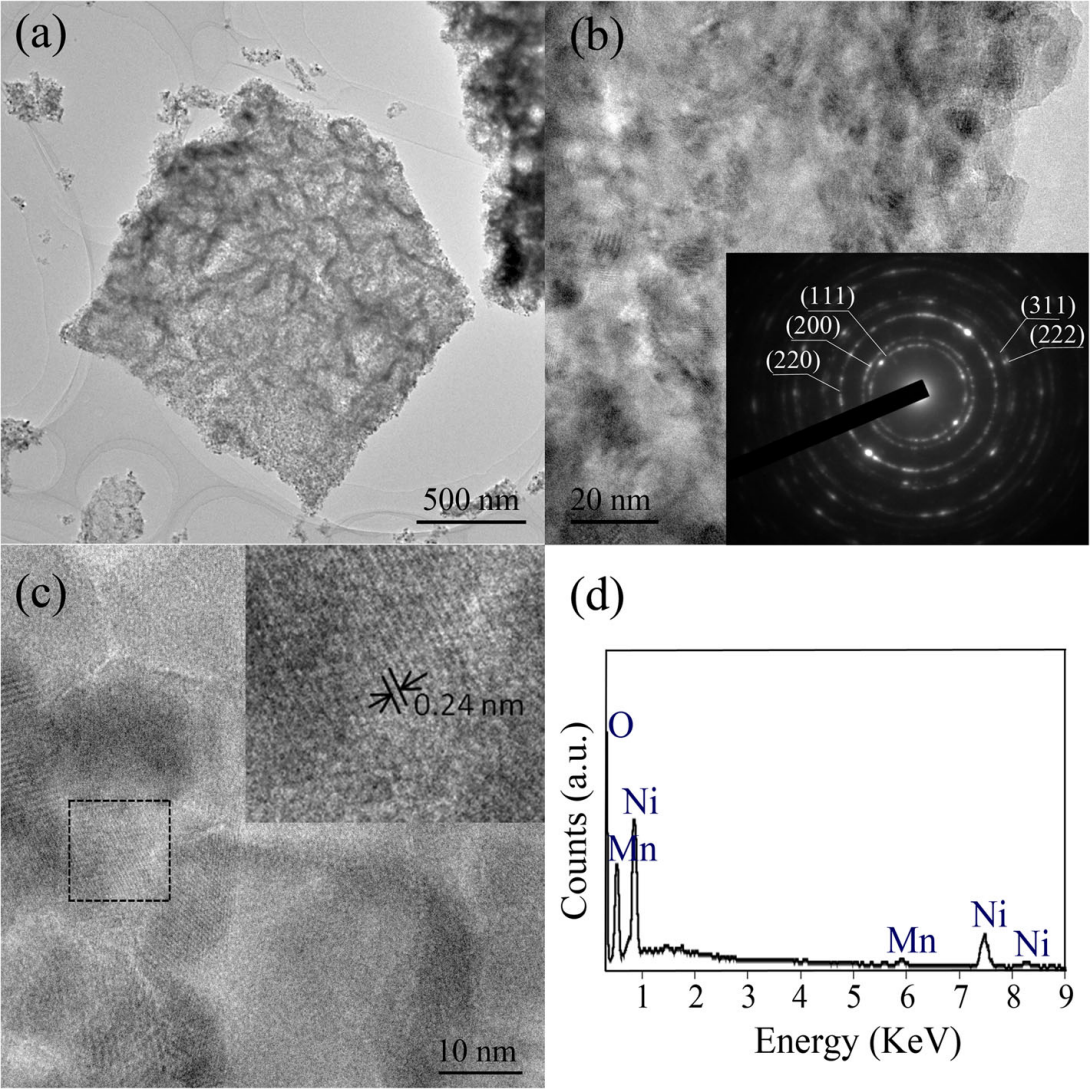
**a**–**c** TEM and HRTEM images of the hybrid nanoflake peeled from CC/NiO/MnO_2_ electrode, with the corresponding SEAD pattern shown as the inset of Fig 4b. **d** Local EDS analysis of the nanoflake

Figure 4a shows a typical transmission electron microscopy (TEM) image of an individual NiO/MnO_2_ nanoflake in which a highly porous but continuous structure could be found. The higher-magnitude TEM shown in Fig. 4b demonstrates that the nanoflake is composed with crystalline and amorphous particles, which could correspond to NiO core and MnO_2_ shell respectively, and the porous structure is also confirmed. These generated pores could be due to the loss of water from the structure during annealing process of nickel hydroxide to form porous “root”, and then the self-limiting process is conducted based on the porous “root” to make MnO_2_ shell interpenetrate into porous NiO. The corresponding SAED pattern showing as an inset of Fig. 4b suggests the polycrystalline nature of the NiO core. The diffraction rings are respectively assigned to (111), (200), (220), (311), and (222) planes, which are in good agreement with the above XRD results. The SAED pattern can also indicate the amorphous nature of the shell, in which no other diffraction peaks were observed besides those from NiO. This judgment could be verified by the random atom arrangement of the shell in HRTEM (Fig. 4c). The enlarged HRTEM image of the core in the rectangular area of Fig. 4c is shown in its inset. The lattice spacing of 0.24 nm could be indexed to the (111) crystal planes of the cubic NiO phase, which further confirms the formation of crystalline structure of NiO nanosheets. Local EDS analysis for the obtained nanoflake is shown in Fig. 4d, in which Mn, Ni, and O elements are detected, confirming the NiO/MnO_2_ composite structure.

**Fig. 5:**
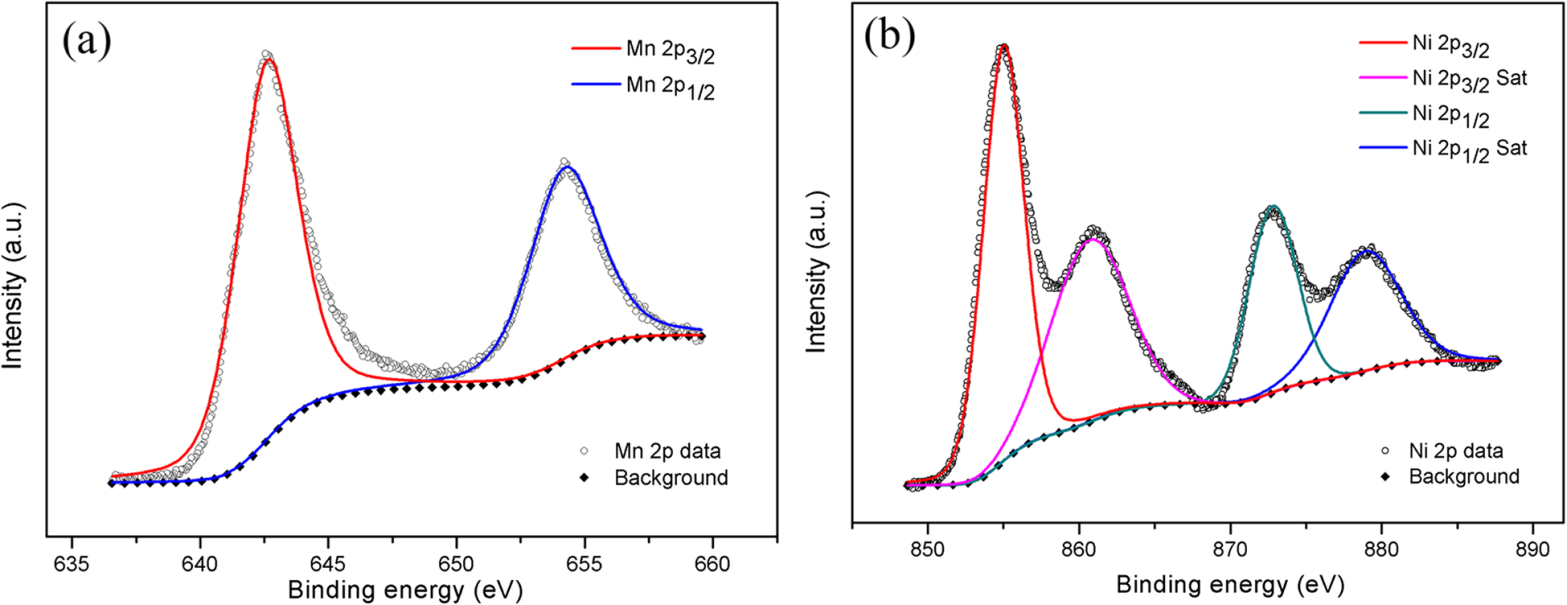
XPS spectra of **a** Mn_2p_ and **b** Ni_2p_ for NiO/MnO_2_ electrode

To further explore the chemical information of the as-prepared heterosturcture, XPS has been employed to analyze the chemical bonding within the nanoflakes, where monochromatic Al Kα X-ray source was operated at 150 W. XPS data (Fig. 5) showed that Mn 2p_3/2_ and Mn 2p_1/2_ peaks were located at ca. 642.6 and 654.2 eV, suggesting the element Mn in the sample was present in the chemical state of Mn^4+^. The Ni 2p_3/2_ and Ni 2p_1/2_ lines were found at the binding energies of about 855, 860.9, 872.8 and 879 eV, indicating the existence of NiO.

Because of the good connection between the active materials and substrate, the obtained CC/NiO/MnO_2_ hybrid structure can be directly used as a binder-free electrode for SC to estimate its electrochemical properties. Some basic parameters for the electrodes were measured by cyclic voltammograms (CV), galvanostatic charge/discharge (GCD), and electrochemical impedance spectroscopy (EIS).

**Fig. 6:**
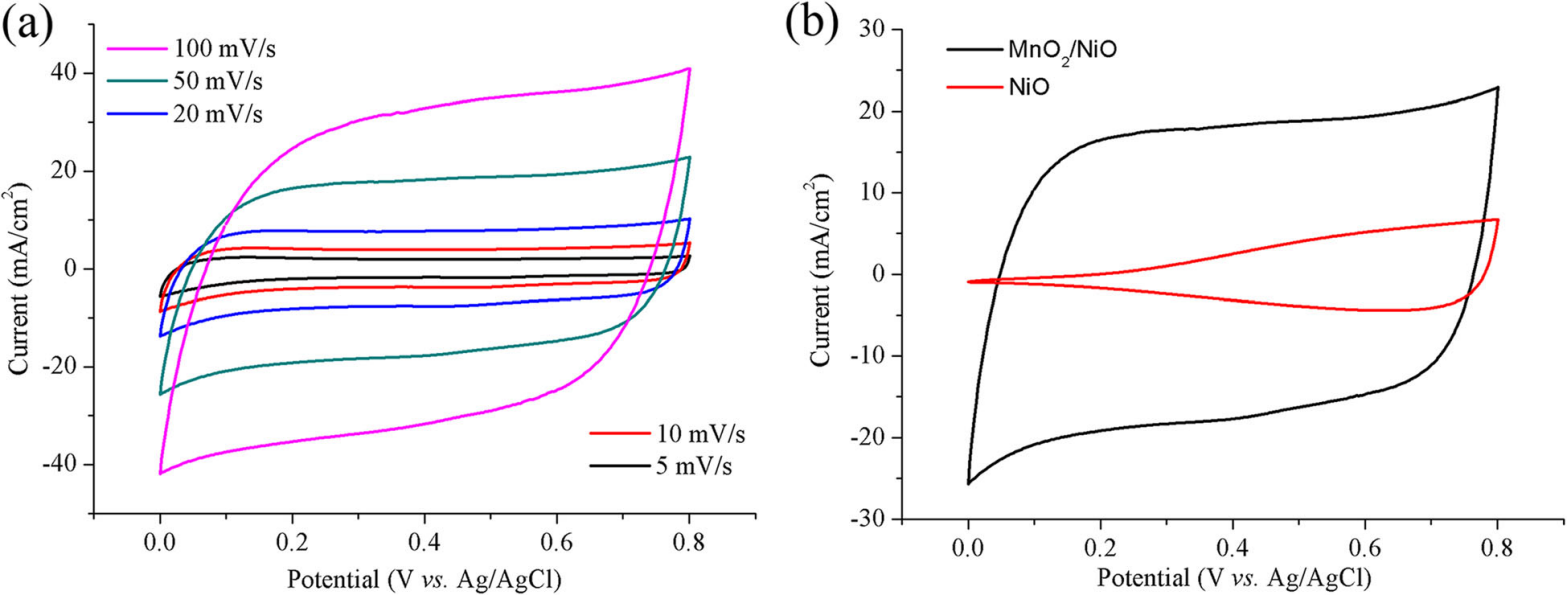
**a** CV curves of CC/NiO/MnO_2_ electrode at different scan rates. **b** CV curves of CC/NiO and CC/NiO/MnO_2_ at a scan rate of 50 mV/s

To test the electrochemical capacitive performance of the NiO/MnO_2_ nanoflakes grown on carbon cloth, CV measurements were conducted in 1 M Na_2_SO_4_ solution with Ag/AgCl and platinum foil as the reference and counter electrode, respectively. The CV was firstly recorded at different scan rates from 5 to 100 mV/s (Fig. 6a), and CV of pristine NiO nanosheet array is also shown for comparison (Fig. 6b). For NiO/MnO_2_ hybrid structures, the CV curve exhibits a near symmetric rectangular shape and exhibit near mirror-image current response on voltage reversal, which indicates that these structures have good capacitive behavior. Moreover, it can be apparently observed that CV curves of the hybrid electrode are closer to rectangle at lower scan rates (from 5 to 20 mV/s), while the curves deform seriously with the scan rate increasing. This deformation could be attributed to the kinetically limited pseudocapacitive reactions and diffusion limited charge transport in the MnO_2_ film.

For the pristine NiO nanosheet integrated CC, the CV demonstrates distinct difference, as shown in Fig. 6b. The CV integrated area of pristine NiO array is apparently smaller than that of NiO/MnO_2_ array at the scan rate of 50 mV/s, which means decreasing pseudocapacitance. The specific geometric capacitance (C_A_) values can be calculated based on the CV results, according to the following equation:

$$ {C}_A=\frac{{\displaystyle \int } i(v) dv}{2 SV} $$

where *S* is the potential sweep rate (V/s) and *V* represents the potential range. The specific capacitance of the NiO/MnO_2_ composite is approximately 6 times greater than that of the NiO (316.37 vs. 52.85 mF/cm^2^). The improved C_A_ of the hybrid array may result from the synergistic effect of MnO_2_ and NiO integrated into an ordered structure directly on current collector. Firstly, the two kinds of excellent pseudocapacitive materials densely cover on the entire surface of carbon fiber without using additives thus to provide more active spaces and improved electron transfer pathways. Secondly, the prepared thin MnO_2_ shell grown on NiO nanosheets are interpenetrated and form a highly porous structure (as shown in Fig. 4), allowing an effective ion exchange between the electrolyte and the two active materials, which is in favor of the fast redox reaction to obtain high specific capacitance; all these characteristics assure plenitudinous contact between the electrolyte and the active materials and thus to accelerate ion diffusion in redox reactions. Considering the loading mass of MnO_2_ is 1.29 mg, the specific gravimetric capacitance is calculated to be 204.3 F/g.

**Fig. 7:**
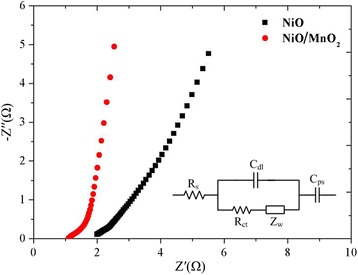
Impedance Nyquist plots of the electrodes, with the corresponding equivalent circuit model shown as the *inset*

In addition, the electrochemical behaviors of NiO and NiO/MnO_2_ electrodes have also been characterized with EIS measurements, and their Nyquist plots are shown in Fig. 7. The NiO/MnO_2_ electrode has nearly vertical linear shape that could be resulted from sufficient wetting of the internal surface of porous electrode by the electrolyte. Furthermore, no evident semicircle corresponding to charge transfer resistance could be found, which indicates a negligible charge transfer resistance. EIS data were analyzed by fitting the experimental data in accordance to the equivalent circuit model as shown in the inset of Fig. 7, with the fitted results showing in Table 1. The equivalent circuit model consists of a combined resistance *R*_s_ (the sum of ionic resistance of electrolyte, the intrinsic resistance of active material, and contact resistance at the active material/current collector interface), a charge transfer resistance *R*_ct_, Warburg resistance *Z*_w_, double-layer capacitance *C*_dl_, and pseudocapacitance *C*_ps_ [27]. From the fitted data, it can be found that the *R*_s_ of NiO/MnO_2_ electrode is around 1.367 Ω, which is much lower than that of the NiO electrode (1.974 Ω), indicating the improved charge transport properties of the NiO/MnO_2_ electrode. What’s more, *C*_ps_ is magnitudes 2-3 lager than *C*_dl_ for both NiO and NiO/MnO_2_ electrodes, revealing that the capacitance of both the electrodes mainly contributed from Faradaic supercapacitance. On the other aspect, *C*_ps_ and *C*_dl_ of NiO/MnO_2_ electrode are enormously enhanced compared with that of NiO electrode, mainly due to the increased amount of electrochemical active material (MnO_2_), and thus providing more active area for electrochemical reaction. Moreover, with the coverage of MnO_2_ shell, the ion diffusion of the electrode is also improved greatly, shown in the Table 1 as the value of *Z*_w_ is much higher for NiO/MnO_2_ electrode than NiO electrode, which could be resulted from the ultrathin morphology of MnO_2_ shell [23]. All the results suggest that the electrode has very small resistance with good ion response at high frequency ranges, indicating that the obtained CC/NiO/MnO_2_ structure could indeed act as a good SC electrode.

**Table 1:** Impedance parameters of the carbon cloth/NiO/MnO_2_ electrode

Sample	*R*_s_	*C*_dl_	*R*_ct_	*Z*_w_	*C*_ps_
NiO	1.974	7.788 × 10^−5^	0.2282	0.04875	0.05397
NiO/MnO_2_	1.367	2.65 × 10^−3^	0.2055	0.3471	0.3282

**Fig. 8:**
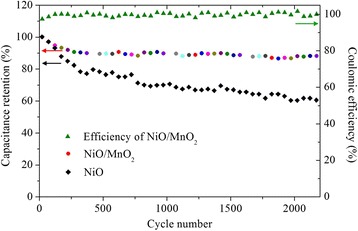
Cycling performance of CC/NiO/MnO_2_ and CC/NiO electrode, and Coulombic efficiency of the CC/NiO/MnO_2_ electrode up to 2200 cycles at the current density of 10 mA/cm^2^

Cycling performance is also an important property for on-chip SCs. The cycling test of the NiO/MnO_2_ hybrid electrode was carried out at the current density of 10 mA/cm^2^ in 1 M Na_2_SO_4_ electrolyte, as shown in Fig. 8. After 2200 charge-discharge cycles, the capacitance retains around ~89% of the original value with >97% Coulombic efficiency at the whole measuring range. The pristine NiO array, however, has 39% capacitance loss after cycling. From the different capacitance retention of NiO and NiO/MnO_2_ electrodes, it is believed that the NiO/MnO_2_ electrode possesses a more superior cycling stability than NiO electrode. This improvement could be due to that MnO_2_ nanoflakes share intimate “root contact” with NiO nanosheets and at the same time fill the networking voids of NiO array. The flexible interpenetrated MnO_2_ nanoflakes can help to maintain the structural integrity and mechanical adhesion with current collector and thus benefit long-term electrochemical cycling.

**Fig. 9:**
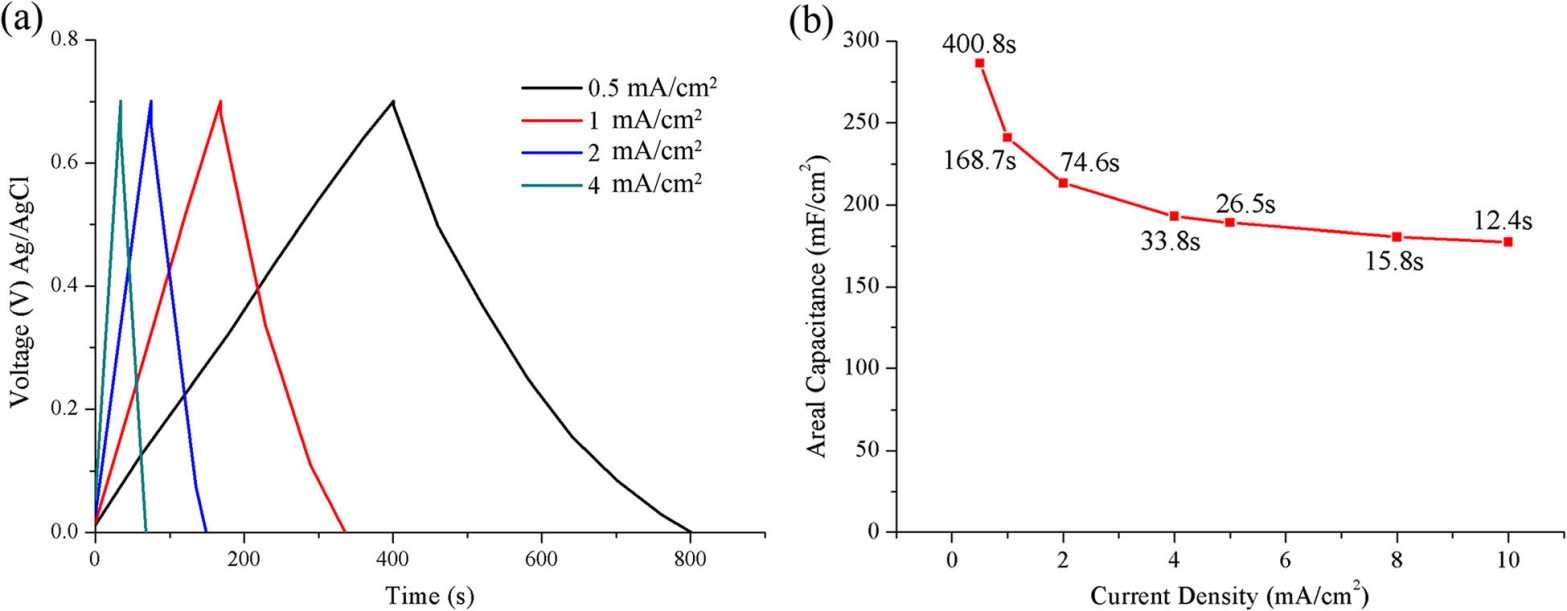
**a** Galvanostatic charging/discharging curves of the electrode at various current densities. **b** Areal capacitance value plot depending on charging/discharging current density

The rate capability of the NiO/MnO_2_ hybrid electrode is also characterized at different current densities, with the galvanostatic charge-discharge curves shown in Fig. 9a, demonstrating good symmetry during the total range of potential. The typical areal capacitance of the NiO/MnO_2_ electrode can be calculated through the following equation.

$$ {C}_A=\frac{I_A\times \varDelta t}{\varDelta V} $$

where ${C}_A$ is the areal capacitance of the electrode, ${I}_A$ is the current density of charge-discharge process, $\mathrm{\triangle} t$ is the time duration after IR drop upon discharge, and $\mathrm{\triangle} V$ is the discharging potential range.

The areal capacitance value vs. current density plot is presented in Fig. 9b, in which the time durations required for each discharging period at different current density are labeled. From Fig. 9b, it’s indicated that the ${C}_A$ decreases apparently as the current density increasing, which may reveal that the reversible redox reaction is a highly diffusion-controlled process. At the current density of 0.5 and 1 mA/cm^2^, the areal capacitance is 286 and 241 mF/cm^2^, respectively. When the charge-discharge rate increases to 10 mA/cm^2^, the areal capacitance decreases to 177 mF/cm^2^, which is about 62% of the areal capacitance at the current density of 0.5 mA/cm^2^. Even the decrement at 10 mA/cm^2^ is relatively high, the areal capacitance of our synthesized electrode is still larger than that of other electrode based on pseudocapacitive materials such as hydrogenated ZnO/MnO_2_@carbon cloth (138.7 mF/cm^2^ at the current density of 1 mA/cm^2^ [8]), α-MnO_2_ nanowires@carbon fabrics, and amorphous Fe_2_O_3_ nanotubes@carbon fabrics (150.0 and 180.4 mF/cm^2^ at the current density of 1 mA/cm^2^, respectively [28]), while how to improve the rate performance of the hybrid structure is our future work.

## Conclusions

In summary, NiO/MnO_2_ core-shell nanoflakes were successfully integrated on flexible CC mainly based on two-step procedure including hydrothermal and self-limiting process. The proposed synthesis route is general, low cost, and scalable for large-scale production, and also, it can be easily extended to synthesize various functional hybrid nanostructure arrays. The electrochemical performance of such a hybrid structure as supercapacitor electrode was systematically explored. The results show that with the introduction of MnO_2_ thin layer, the electrochemical performance of CC/NiO electrode has been greatly improved. Furthermore, the CC/NiO/MnO_2_ electrode exhibits much higher areal capacitance compared with many directly-grown pseudocapacitive nanostructure films. It is indicated that the high-performance 3D hybrid structures have promising applications in flexible supercapacitors.
